# Developing a Digital Technology System to Address COVID-19 Health Needs in Guatemala: A Scientific Diaspora Case Study

**DOI:** 10.3389/frma.2022.899611

**Published:** 2022-07-22

**Authors:** Juan Roberto Alvarado, Ximena Lainfiesta, Alejandra Paniagua-Avila, Gabriela Asturias

**Affiliations:** ^1^Fundación Desarrolla Guatemala para la Educación y la Salud, Guatemala City, Guatemala; ^2^Epidemiology Department, Mailman School of Public Health, Columbia University, New York, NY, United States

**Keywords:** scientific diasporas, COVID-19 pandemic, technology, chatbot, Guatemala, capacity building, brain circulation

## Abstract

Scientific diasporas are organized groups of professionals who work together to contribute to their country of origin. Since the start of the COVID-19 pandemic in 2020, scientific diasporas around the world have focused their efforts to support the public health response in their countries of origin. As the first cases of COVID-19 were reported in Guatemala in March of 2020, a team of four Guatemalan nationals, residing abroad and in-country, started collaborating to tackle COVID-19 misinformation and issues with healthcare services navigation. Their collaboration was facilitated by FUNDEGUA, a Guatemalan nonprofit, which provided a legal framework to establish partnerships and fundraise. The team created a digital technological system called ALMA (*Asistente de Log*í*stica Médica Automatizada* in Spanish). A female character named ALMA was created to personify the digital information services, through social media profiles, an interactive website, a free national multilingual call center, and an artificial intelligence-based chatbot. More members joined the nascent interdisciplinary diaspora through professional/personal references or social media. ALMA provided a platform for Guatemalan nationals to contribute with their skillset to their country during a global crisis through flexible schedules and short- or long-term involvement. As the team grew, the services for query resolution and information dissemination expanded as well. The ALMA initiative shows that scientific diasporas can provide an avenue for professionals to contribute to Guatemala, regardless of their residence and job commitments.

## Introduction

Scientific diasporas are networks of professionals who reside abroad, have had experience working or studying abroad, and strive to contribute to their country of origin through their work in science, technology, or engineering industries (Séguin et al., 2006). Most diaspora members are citizens of low- and middle-income countries (LMICs) and live in the G-20 nations as these countries host more than 60% of the migrants worldwide (Beirens et al., 2021). Diaspora members contribute, in the form of remittances, transfer of knowledge, and innovations (Beirens et al., 2021), providing LMICs with access to highly skilled workers (Séguin et al., 2006). While highly skilled migrants and diaspora members are often motivated to contribute to their country of origin, they face several barriers, such as time constraints, financial hurdles, poor infrastructure for knowledge transfer, unclear needs, or requests from their country of origin to contribute, and concerns that their lack of seniority in their field could pose a challenge to their credibility as a project leader (Séguin et al., 2006).

On the 13th of March 2020, the World Health Organization declared the novel coronavirus 2019 (COVID-19) a pandemic (World Health Organization, 2020). By then, COVID-19 had spread around the world with over 400 million confirmed cases (World Health Organization, 2020). To deal with the ongoing pandemic, researchers across the world raced to develop a vaccine. By May 2020, approximately 115 COVID-19 vaccine concepts were being developed and a few (5–6) were in human clinical trials (Ciotti et al., 2020). The Food and Drug Administration (FDA) approved the first COVID-19 vaccine for emergency use in December 2020 (US Food Drug Administration, 2020). As of today, over 1 billion vaccine doses have been administered (World Health Organization, 2022).

Over the last decade, digital and mobile technologies have revolutionized multiple industries and transformed many aspects of daily life (Budd et al., 2020). This transformation became even more apparent during the COVID-19 pandemic when digital technological solutions, which could be deployed faster and at larger scales than manual solutions, were necessary for efforts to contain the virus (Whitelaw et al., 2020). The rapid growth in the mobile technology sector was one of the determining factors in the fast democratization of these technological solutions (Budd et al., 2020). In 2019, 67% of the global population subscribed to mobile devices (Budd et al., 2020). Since the start of the pandemic, digital innovations have been used to manage and respond to the COVID-19 crisis. Applications have included disease screening, contact tracing, quarantine and self-isolation, and clinical management software (Whitelaw et al., 2020).

The novelty and threat of COVID-19 led to the rapid development of large amounts of scientific evidence and unchecked information. Public communication of accurate and updated scientific evidence became a challenge. Based on this and fueled by their desire to give back to their country, two Guateleman engineers residing in Guatemala proposed to create an artificial intelligence-based chatbot that could answer COVID-19 questions from multiple people at once, and that could be updated as the new scientific evidence arose. They partnered with two Guatemalan scientists with clinical and epidemiology expertise who were residing abroad. Together they decided to implement this idea capitalizing on their membership with FUNDEGUA (in Spanish, the abbreviation stands for Fundación Desarrolla Guatemala para la Salud y Educación), a Guatemala-based foundation founded by two Guatemalans and a Duke University professor in 2015. FUNDEGUA's goal is to channel its academic, network, and financial resources to support research-based development initiatives in Guatemala. The foundation was inaugurated with a chronic child malnutrition project, funded by the Guatemalan Tigo Foundation and Duke University, and through Duke students led a capacity-building effort of Tz'utujil (Mayan) scientists and local health promoters, who continued the research and implementation efforts. Since then, FUNDEGUA has spearheaded evidence-based healthcare and education projects that foster collaborations between Guatemalans residing abroad and in-country, leading up to the creation of the ALMA (which stands for its Spanish full name, Asistente de Logística Médica Automatizada) initiative. The non-profit offered staff and resources to support the development of this new idea.

In this article, we describe the consolidation of an interdisciplinary diaspora of Guatemalans, residing in-country and abroad, who developed the digital health system ALMA founded and implemented as a public-private partnership between a Guatemalan non-profit and Guatemala's public health system. This diaspora solidified as its members came together to contribute to the pandemic response by creating a system to disseminate evidence-based COVID-19 information at scale to prevent and manage COVID-19 at the individual and community level. We describe the professional backgrounds, motivations, and organization of team members to join this effort supported by the existing Guatemalan non-profit FUNDEGUA. Finally, we analyze the successes and limitations of how this diaspora was formed and discuss the lessons learned so they can serve as examples for other global diasporas in LMICs.

## Methods

### Setting

ALMA was developed by a scientific diaspora of Guatemalans. Guatemala is a Central American country with 18 million people, half of whom are Maya-indigenous. In addition to Spanish, there are 22 indigenous languages. While the nation was recently categorized as an upper-middle-income country, this economic indicator masks wide social and economic inequalities and slow progress in human development indicators (Class et al., 2020). In fact, Guatemala lags behind the Latin American region in terms of human development (Class et al., 2020). With one of the lowest public health investments (1% of the gross domestic product) and one of the lowest densities of healthcare workers (12.5 per 100,000 population) in the region, Guatemala's public health system was ill-prepared to respond to the COVID-19 pandemic (Avila et al., 2015; CEPAL, 2020). While the public health system is meant to provide universal coverage (Becerril-Montekio et al., 2011), even before the COVID-19 pandemic (1995–2012), out-of-pocket healthcare spending consistently represented over half of the total health expenditure (Class et al., 2020). In contrast, in Guatemala subscriptions to mobile devices are above 110 per 100 people since 2015 (World Bank, 2022), although this number does not reflect potential disparities between rural and urban populations.

After the first case of COVID-19 was detected in Guatemala on March 13, 2020, multiple surges of COVID-19 have overburdened the healthcare system, leading to more than 600,000 registered COVID-19 infections (Garcia, 2021), more than 16,000 confirmed deaths (Garcia, 2021) by February 2022, and significantly increased excess mortality rates compared to previous years (Martinez-Folgar et al., 2021). Most public health information about COVID-19, including vaccinations, has been disseminated in Spanish. Vaccination efforts have been slow, with estimations showing that by February 2022, about 30% of the population had received two doses, one of the lowest coverages in the Latin American continent (Johns Hopkins, 2022). The distribution of COVID-19 vaccines has been characterized by marked inequalities, with reports showing that more than 65% of Guatemala City's population have received two vaccination doses, compared to less than 30% among indigenous populations living in the rural departments (Lab de datos, 2022).

### Data Collection

FUNDEGUA's administrative records were used to describe the members of this scientific diaspora who contributed to the creation of ALMA. This data is collected every time someone joins the team and is renewed annually by the administrative team through a Google Form survey. Approximately five volunteers did not reply to the latest survey and were excluded from the analysis. The database provided by FUNDEGUA was de-identified (names, addresses, emails, and phone numbers were removed) and exported in csv format. The following variables were shared: gender, age, Guatemalan department of origin, highest academic degree, area of specialization (studies), countries where they have experience studying in, role within ALMA, the initial connection to ALMA, motivations for joining the team, prior experience in social impact projects, Guatemalan departments they have experience working in, and country they were residing in when they joined. We also utilized administrative data from meeting minutes to identify and describe the principles, partnerships, strategies objectives of the ALMA initiative.

### Data Analysis

The database was cleaned and aggregated using Python (Van Rossum and Drake, 2009). The raw data output had flattened responses, meaning that questions that allowed for multiple responses had a column per possible response, each column name equal to the answer choice. Single response questions had a single column with the corresponding answer. We used the Pandas (McKinney, 2010) Python package to import the csv file into a data frame format. We split each question into two data frames, single response questions and multiple response questions. For single response questions, each answer was summed and divided by the total answers to obtain the response percentage. For multiple response questions, a data frame including the columns which represented the questions' responses was created. Then each column was summed and divided by the total to obtain the percentage. Finally, each data frame was shared to create the output table using Microsoft Excel.

### Data Visualization

The principles, partners, strategies, and objectives were portrayed in a Figure using Adobe Illustrator 2020. The distribution of team members across the world based on where they studied and where they resided in before joining the project was portrayed through a chloropleth global map generated in Datawrapper and further edited using Adobe Illustrator 2020. The Guatemalan departments where the team members had experience working in prior to joining ALMA were depicted using a chloropleth Guatemala map generated in Datawrapper.

## Results

### ALMA Initiative

**Figure 1:**
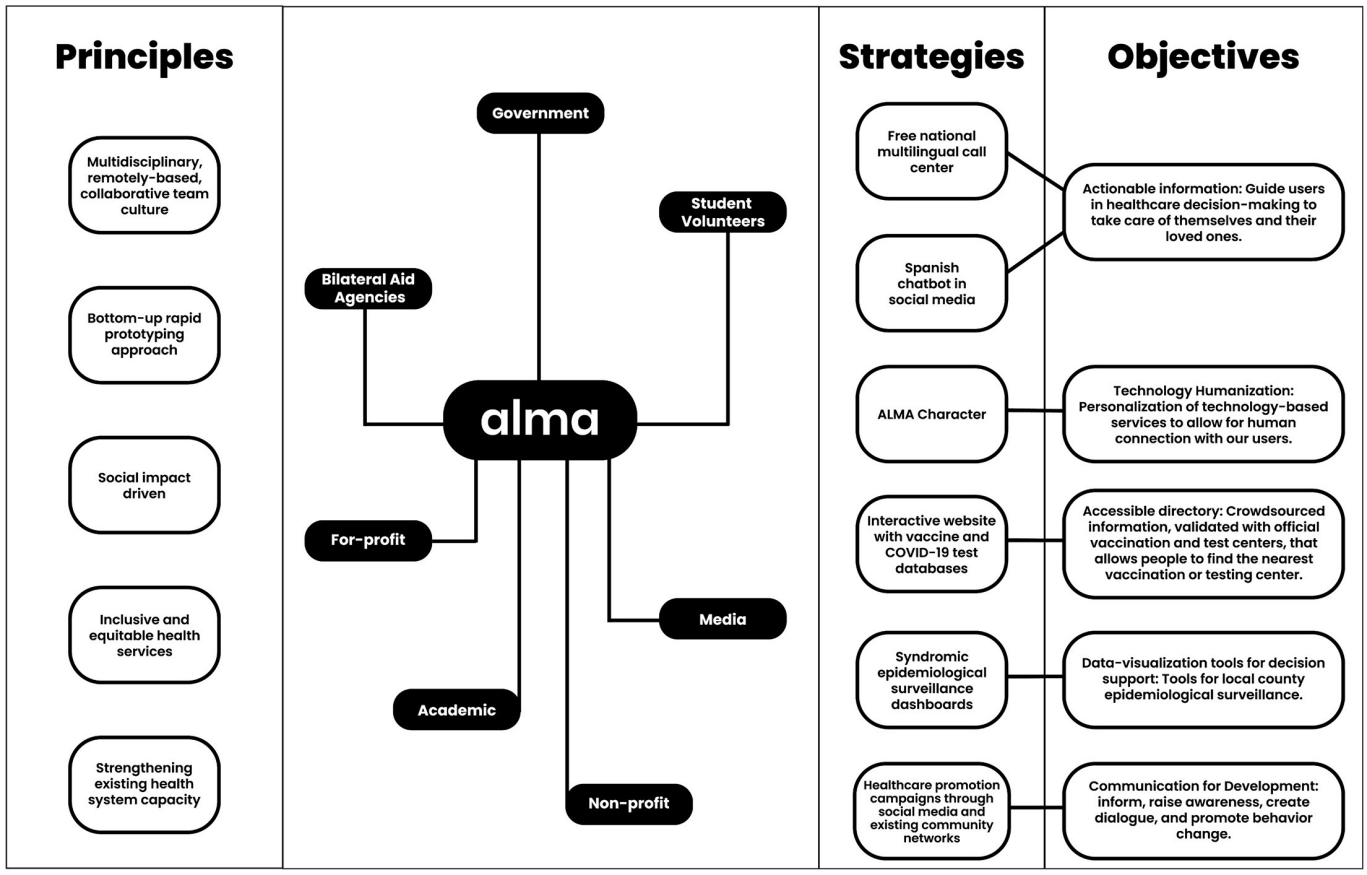
Principles, partners, strategies, and objectives. Principles guide the internal and external collaborations; partners contributed to the strategies implemented to reach the objectives. Bilateral Aid Agencies: CDC, BID Lab, BID Guatemala. Academic institutions: Universidad del Valle de Guatemala, Laboratorio de Datos. Government institutions: Ministry of Health and Social Assistance, National Secretariat of Science and Technology, Vice Presidency, Ministry of Education, Plan Trifinio. Non-profit organizations: Asociación de Salud Integral, Volunca, Wuqu'Kawoq-Maya Health Alliance, FUNDESA, La Ruta. Media: Agencia Ocote, Publinews, Radio Infinita, Radio La Mega, Diario de Centro América, La Red. For-profit organizations: Tecniscan, Allied Global, Pronto BPO. Student Volunteers from: Universidad de San Carlos, Universidad de Rafael Landivar, Universidad Francisco Marroquín, Georgetown University, Universidad del Valle de Guatemala, George Washington University, Notre Dame University.

Figure 1 describes the principles that guided the creation of ALMA, the collaboration between multiple partners, and the strategies employed to reach the key objectives of this initiative. Results on the implementation and impact outcomes of ALMA will be reported in a separate publication.

The ALMA initiative is founded on principles (see Figure 1) that enabled collaborations between members residing abroad or in-country to leverage technology in response to the global problem of COVID-19 misinformation. ALMA is backed by a multidisciplinary (see Table 1) and collaborative team, that worked remotely, respecting social distancing protocols, and enabling participation from members residing in different countries. The team focused on rapidly prototyping different approaches to the misinformation problem, getting feedback directly from end-users for future iterations. The work was driven by social impact and focused on improving access to available health services in Guatemala. Lastly, the team focused on strengthening existing health system capacities by working alongside the government's efforts to address the needs of the COVID-19 pandemic.

**Table 1:** Key characteristics of ALMA team members.

**Characteristic (*n =* 29)**	**Percentage**
Gender	
Female Male	48 52
Current age (ranged from 19–38)	
19–22 23–26 27–30 31–34 35–38	14 38 31 10 7
Guatemalan department of origin	
Guatemala Chiquimula Sacatepéquez Jutiapa	91 3 3 3
Highest academic degree	
High School Bachelor Master Doctorate	10 52 34 3
Area of specialization *	
Technology Health Communication Other Politics Administration Education	28 24 24 17 10 10 3
Role within ALMA *	
Technology Clinical Communication Customer service Administration Management Education	28 24 24 24 10 10 7
Initial connection to ALMA*	
Personal/professional reference Social media post Job posting Other	83 24 3 3
Prior experience in social impact projects	
Yes No* Lack of awareness of opportunities Lack of time Conflict with prior job/commitments Other	66 34 60 20 10 10

*Information was obtained from ALMA's administrative records. Percentages are reported. Data items marked with an asterisk indicates that participants had the option to select multiple answers for those questions*.

Partners provided guidance and mentorship in the rapid prototyping phases, established collaborations legitimizing the nascent initiative, and provided funding to pilot, implement, and scale their impact (see list of partners in Figure 1). Bilateral aid agencies provided funding through all stages of development. Academic institutions helped by providing technical expertise. Working with government institutions was essential to scaling their impact across larger segments of the population. The Ministry of Health and Social Assistance was instrumental in providing the required endorsement for ALMA to work with public health services and providers. Non-profits enabled the ALMA initiative to establish local partnerships and introduce its services outside of urban centers to underserved, indigenous populations. Media outlets helped by providing free and wide dissemination of the services provided by ALMA to the population. Guatemalan student volunteers from U.S. and Guatemalan universities contributed through short-term experiences with their technical expertise.

The ALMA initiative employed strategies to accomplish key project objectives. The team first focused on accomplishing objective 1, providing actionable healthcare information to guide users in their decision-making, for example: when to get a PCR or antigen COVID-19 test based on exposure date, booster eligibility based on primary schedule, latest country guidelines for quarantine after exposure, among others. This information was disseminated by an artificial intelligence-based chatbot in Spanish through social media accounts and a free national multilingual (Spanish and five Mayan languages) call center in coordination with national and local healthcare authorities. To establish trust with users, the team personalized the technology employed to deliver the informational services with a friendly character. The character was the result of internal workshops to define qualities that embodied the service the team wanted to provide its users. Additionally, as part of the national vaccination efforts, ALMA created the website www.vacunasgt.com which contains updated information regarding vaccination centers' locations, eligibility requirements, schedules, and wait times. The information is updated through triangulation of official government information and crowdsourcing of live reports from users, volunteers, and staff at vaccination centers. The data collected through ALMA's different channels, such as suspected cases identified through syndromic surveillance, was aggregated, and visualized through dashboards for healthcare authorities, as a support for their decision-making. Lastly, the social media profiles of ALMA were used as mediums to disseminate healthcare promotion campaigns to promote behavior change.

### ALMA Multidisciplinary Team

ALMA's team includes members specialized in digital technology, clinical medicine, communication, customer service, business administration, management, and education. ALMA's administrative information shows that the entire team is composed of Guatemalan nationals, aged 19–38 years, the majority born in Guatemala City and highly educated (see Table 1). The majority were recruited to join ALMA, either through personal/professional relationships with current team members/co-founders or through ALMA's presence on social media. Lastly, most members (66%) have had prior experience in social impact projects in Guatemala. The 34% of the team members who didn't have prior experience have identified barriers such as lack of awareness of opportunities, lack of time, and conflict with prior job/commitments (see Table 1).

ALMA's team members have reported multiple enablers for joining this scientific diaspora while working on another full-time job and residing abroad or in-country. Enablers include: 1) a platform that allowed short-term participation with the possibility for a large impact amplified by an established team, 2) a Guatemalan foundation that backed the project providing a legal framework for its execution and fundraising, 3) the possibility to work remotely in a flexible environment, allowing members to contribute to their country of origin.

**Figure 2:**
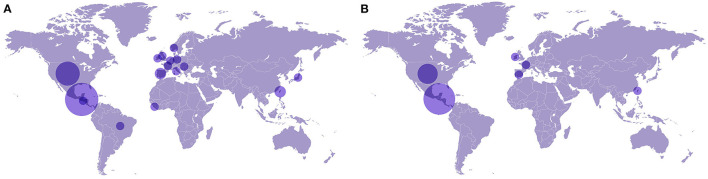
Distribution of the countries ALMA team members have experience studying and residing in. **(A)** shows the countries where team members have studied. **(B)** shows the countries where the team members resided when they joined the ALMA team. The larger circle size represents a larger number of team members for each country.

ALMA team members have a diverse set of experiences and training abroad and in-country, with more than half studying outside of Guatemala, the top three nations being three income countries, the United States, Taiwan, and Spain (see Figure 2A). As displayed in Figure 2B, 62% resided in Guatemala when they joined ALMA, 24% in the United States, 3% in Spain, 3% in France, 3% in Ireland, and 3% in Taiwan. Currently, 91% of the team members are residing in Guatemala, 3% in the United States, 3% in Spain, and 3% in Canada. As the initiative advanced, there was a shift from members residing abroad to in-country (62 to 91%), mostly attributed to the COVID-19 pandemic motivating members to be closer to their family during uncertain times, job security, and education opportunities abroad being shut down.

**Figure 3:**
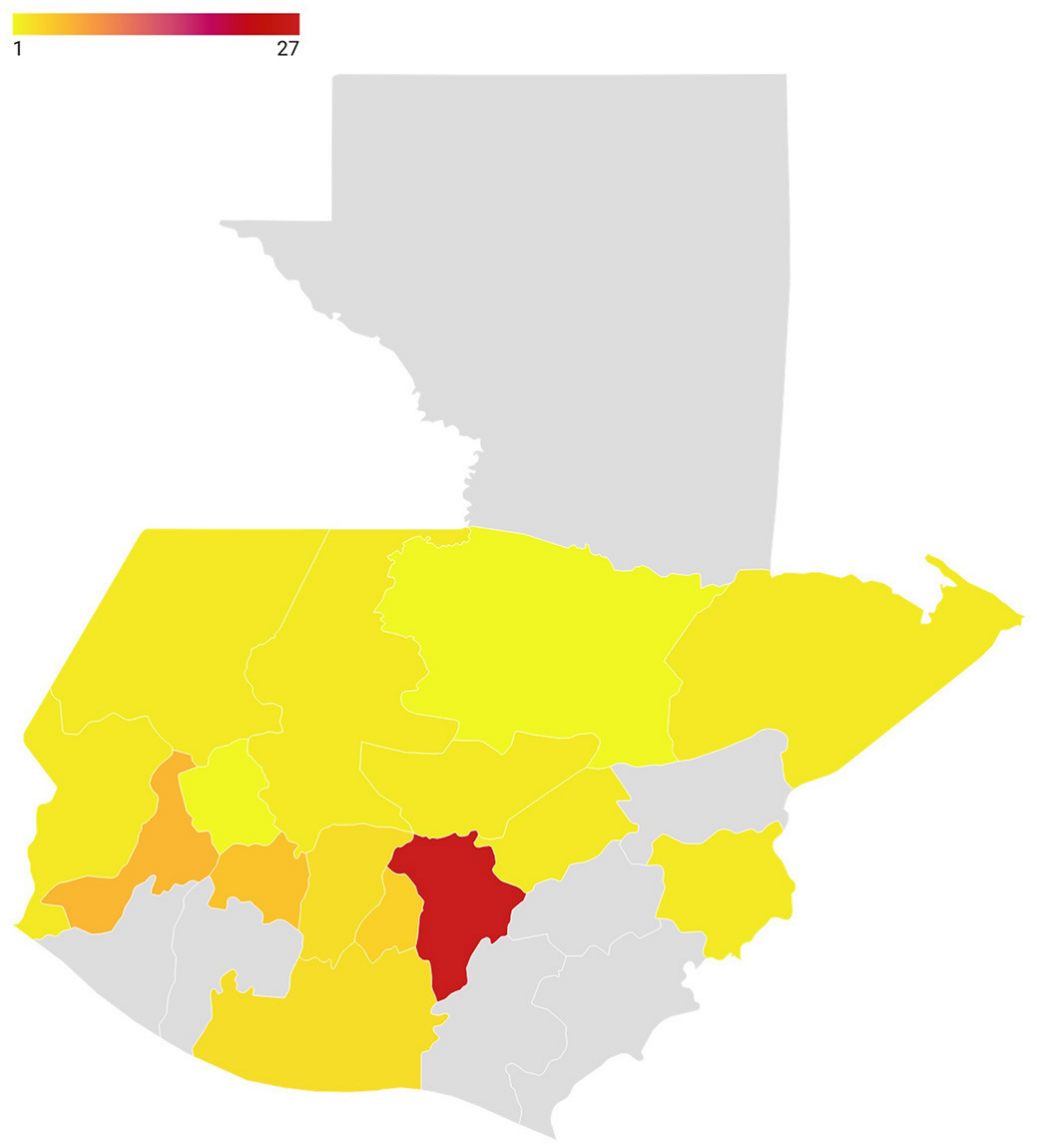
Guatemalan departments where ALMA team members have work experience. Some team members have had experience in more than one department. The minimum was 1 person and the maximum was 27 people with experience working in a particular department. A darker red color corresponds to the maximum number of team members (*n* = 27), and a lighter yellow color to the minimum (*n* = 1). Gray indicates that none of the team members have had work experience in that department.

Efforts were made to recruit people residing in the country because it was important to have availability to travel to rural locations to better understand the misinformation problem in Guatemala. Figure 3 shows the experience ALMA team members had working in different Guatemalan departments (15 out of the 22 are represented), which was crucial for the implementation of the project outside urban centers and the capital city.

## Discussion

The ALMA initiative showcases an example of the consolidation of an interdisciplinary diaspora of Guatemalans residing abroad and in-country at the start of the COVID-19 pandemic. Founded upon a loosely connected group of computer engineers and physician-scientists and facilitated through an existing non-profit organization, the diaspora behind ALMA solidified to tackle the global problem of COVID-19 misinformation. Based on successful cases in other countries in Latin America (Panamá, México, Colombia, Brazil) (Albert Einstein - Sociedade Beneficente Israelita Brasilera., 2020; El Universal., 2020; Ministerio de Salud de Colombia., 2020; Ministerio de Salud de la República de Panamá., 2020; Secretaría de Salud de México., 2020), the team created its first minimum viable product, a chatbot, with the following objectives 1) information delivery about COVID-19 symptoms, management, and feasible prevention practices, and 2) first-line triage to differentiate high and low-risk patients based on reported symptoms, epidemiological risk (travel and exposure history) (Lifespan., 2020; Patel et al., 2020; Smith and Boslett, 2020).

The global COVID-19 crisis spurred collaborations among migrants and diasporas around the world to respond to the crisis and support their families and communities “back home” (International Organization for Migration, 2020). On one hand, rapid and significant changes to the global landscape have tested diasporas' capacity to continue fostering development and innovation in their countries of origin (Beirens et al., 2021). Reports have described COVID-19-related factors, such as reduced employment opportunities in the G-20 countries and limited mobilization between countries, as potential threats to diasporas' sustainability and capacity to continue collaborating with their countries of origin (Beirens et al., 2021). Simultaneously, members of diasporas have reacted by supporting and connecting with each other and their families and communities back home, proving their resiliency, creativity, and reaction capacity to continue transferring innovations, knowledge, and technology (Beirens et al., 2021). Diasporas have capitalized on existing organizations and associations or created new collaborations to contribute to the response to COVID-19, particularly in the areas of digital technology and health (International Organization for Migration, 2020). Due to the COVID-19 restrictions, collaborations among these diasporas transitioned into online channels, diminishing the barriers of having to travel to meet in person (International Organization for Migration, 2020). This new work-from-home norm allowed the expansion of existing diasporas and the creation of new ones (International Organization for Migration, 2020). The International Organization of Migration developed a report highlighting several diaspora-led initiatives, developed during the COVID-19 pandemic (International Organization for Migration, 2020). In this context, our diaspora of Guatemalans based abroad and in-country emerged to create ALMA. This interdisciplinary group represents technology, communication, social and health science, public policy, and clinical expertise. Members were motivated to join as a contribution to their country of origin during a time of acute need, a possibility to join a group of young and like-minded individuals, for professional development, and because of the flexibility for remote, short-term, or part-time engagement.

Lessons learned from creating this interdisciplinary diaspora include partnering early on with an in-country organization with a track record of supporting similar initiatives and having existing in-country partnerships. This allowed for the rapid development, implementation, dissemination, partnerships, and fundraising for the ALMA initiative. Additionally, providing new members residing abroad or in-country with remote work and flexible schedules, allowed the team to attract talent for either short periods of time or for highly specialized projects within the initiative. A clear social mission that resonated with young professionals, was an instrumental motivating factor for the rapid escalation of the team and for the engagement of diverse partners. The initiative also provided opportunities for university students to join part-time, for their professional development and as an opportunity to give back to their country of origin.

Future considerations include defining a formal recognition or retribution for all of the team members that had short-term engagements in the initiative. This strategy was necessary given that the team was growing rapidly as digital technological solutions were being developed, and there were no clear benefits after concluding your participation within ALMA. Additionally, the initiative started recruiting more members residing in-country with job experience in multiple departments (15 out of 22 departments are represented), to ensure ALMA could transcend beyond the urban centers and capital city, reaching rural and underserved communities. Over time this reduced the efforts within ALMA to engage Guatemalans residing abroad and keep offering opportunities for involvement in short-term specialized projects, which were more emphasized at the beginning of the initiative's creation. More than half of the team members had experience studying and residing abroad prior to joining and 30% moved back to Guatemala throughout their involvement in ALMA.

The ALMA initiative is currently focused on expanding beyond COVID-19, to continue providing reliable, trustworthy, and timely information about personal health, while generating aggregated epidemiological data that can aid decision-making. In partnership with the Ministry of Health and Social Assistance, the initiative provides information on all vaccines, water and foodborne illnesses, and acute respiratory infections. We are working with non-profits and academic partners to expand to non-communicable diseases (malnutrition), vector-borne diseases, and sexually transmitted diseases. This award-winning initiative has been successful at channeling knowledge, abilities, connections, and resources from Guatemalan nationals living overseas partnering with local scientists, engineers, technicians, and public and private institutions. The result has been a network of like-minded individuals that have led to collaborations beyond the ALMA initiative, in education, mental health, and artistic endeavors.

## Data Availability Statement

Administrative datasets were used. Requests to access these datasets should be directed to admin.alma@fundegua.org.

## Author Contributions

GA, AP-A, JA, and XL contributed to the conception and design of the manuscript and wrote sections of the manuscript. JA and GA wrote the first draft of the manuscript, reviewed the administrative records, and built the table. XL created the figures. AP-A contributed substantially to the conceptualization of the manuscript and critically reviewed the final version. All authors contributed to manuscript revision, read, and approved the submitted version.

## Funding

ALMA (Asistente de Logística Médica Automatizada in Spanish) is supported by Fundación Desarrolla Guatemala para la Educación y la Salud (FUNDEGUA) and has been funded in whole or in part through the cooperative agreement between the Centers for Disease Control and Prevention (CDC) and Universidad del Valle de Guatemala (UVG) No GH002243, Interamerican Development Bank Lab project number GU-T1327, and private donors. The authors GA, JA, and XL have received financial compensation from FUNDEGUA.

## Conflict of Interest

The authors declare that the research was conducted in the absence of any commercial or financial relationships that could be construed as a potential conflict of interest.

## Publisher's Note

All claims expressed in this article are solely those of the authors and do not necessarily represent those of their affiliated organizations, or those of the publisher, the editors and the reviewers. Any product that may be evaluated in this article, or claim that may be made by its manufacturer, is not guaranteed or endorsed by the publisher.
